# Midterm Outcomes of BeGraft Stent Grafts Used as Bridging Stents in Fenestrated Endovascular Aortic Aneurysm Repair

**DOI:** 10.1177/15266028221091894

**Published:** 2022-04-26

**Authors:** Rachel E. Clough, Rafaëlle Spear, Justine Mougin, Thomas Le Houérou, Dominique Fabre, Jonathan Sobocinski, Stéphan Haulon

**Affiliations:** 1 School of Biomedical Engineering and Imaging Science, St Thomas’ Hospital, King’s College London, London, UK; 2 Vascular Surgery, CHU Grenoble, Grenoble, France; 3 Aortic Centre, Groupe Hospitalier Paris Saint Joseph, Hôpital Marie Lannelongue, INSERM UMR_S 999, Université Paris-Saclay, Gif-sur-Yvette, France; 4 Vascular Surgery, CHRU Lille, Lille, France

**Keywords:** bridging stent, BeGraft, Bentley, fenestrated, aneurysm, aorta

## Abstract

**Purpose:**

Fenestrated endovascular aneurysm repair (fEVAR) is established for the treatment of juxtarenal, pararenal, and thoracoabdominal aortic aneurysms (TAAAs). Bridging stents are used to connect the main body of the stent graft to the aortic branch vessels. Complications related to the bridging stents compromise the durability of the repair and require urgent re-intervention. Here we present the midterm results of the BeGraft stent graft system used for fEVAR.

**Materials and method:**

All consecutive patients treated with fEVAR and the current BeGraft Peripheral Stent Graft between November 2015 and September 2016 were included.

**Results:**

Thirty-nine consecutive patients (38 men) were enrolled and 101 BeGraft second-generation stent grafts were implanted. The median aneurysm diameter was 60 mm (54.5–67.0 mm). Aneurysms were juxtarenal and pararenal (19/39, 48.1%), type 4 TAAA (3/39, 7.7%), type 1, 2, and 3 TAAA (7/39, 17.8%), type 5 TAAA (4/39, 10.2%), and 15.4% (6/39) had a type I endoleak following a previous EVAR. Fifty-five BeGrafts were implanted in mesenteric arteries (22 in coeliac trunks, 31 in the superior mesenteric artery, and 2 in a hepatic or splenic artery) and 46 into renal arteries (24 right and 22 left). The renal artery diameters were 5, 6, 7, and 8 mm in 9, 7, 26, and 4 patients, respectively. Mesenteric arteries were exclusively stented with 9 and 10 mm diameter devices. The median follow-up was 33 months (IQ25 17–IQ75 36). During follow-up, 11 patients died (28%) from non–aneurysm-related causes. The overall patency rates for bridging stents were 98% and 97% at 1 and 2 years, respectively, with a freedom from secondary procedure rate on BeGraft stent grafts of 96% (97/101). All events occurred on stents implanted in renal arteries.

**Conclusion:**

Early favorable outcomes are confirmed during longer term follow-up. Vigilant surveillance is required.

## Introduction

Endovascular repair is established for the treatment of thoracoabdominal aortic aneurysms (TAAAs) with good short-term and midterm outcomes. Fenestrated stent grafts can be used for this type of repair and require bridging stents to connect the main body of the stent graft to the aortic branch vessels. These branches supply the intra-abdominal organs and maintenance of antegrade flow through these branches is critical to the success of the aneurysm repair and survival of the patient.

Fenestrated endovascular aneurysm repair (fEVAR) has been available more than 10 years, but a dedicated bridging stent is still not available. Instead devices are used off-label as bridging stents, manufactured with a range of designs and materials, and are selected at the discretion of the user. Important complications can occur related to the bridging stents such as occlusion, fracture, dislocation, and endoleak that compromise the durability of the endovascular repair and require urgent re-intervention.

The BeGraft Peripheral Stent Graft (Bentley InnoMed GmbH, Hechingen) is a balloon-expandable covered stent. We have previously evaluated the short-term outcomes of BeGraft stent grafts in fEVAR and demonstrated no bridging stent graft fractures or type Ic endoleaks at a mean follow-up of 13 (11–15) months.^1^ Here we present the midterm results of the BeGraft stent graft system used in fEVAR.

## Methods

### Study Population

All consecutive patients in our center treated with fEVAR (Cook Medical, Bloomington, Indiana) and the BeGraft Peripheral Stent Graft between November 2015 and September 2016 were prospectively enrolled in a database that was retrospectively analyzed. The indication for repair was maximum aortic diameter >55 mm or rapid growth (>10 mm in 12 months). BeGraft stent grafts were used as bridging stents to connect fenestrations to target vessels (renal and mesenteric arteries). The BeGraft device was selected to treat renal arteries if the bridging stent diameter needed was ≥6 mm *or* a length of ≥22 mm was required. It was selected for mesenteric arteries if the bridging stent diameter needed was ≥7 mm in diameter *and* a length of ≤38 mm was required. If a V12/iCast stent graft was not available, a BeGraft was used. Renal fenestrations were 6 × 6 mm and visceral fenestrations were 8 × 8 mm.

Preoperative demographics collected included age, gender, body mass index, American Society of Anesthesiologists (ASA) score, cardiovascular risk factors, estimated glomerular rate filtration (GFR) by Modification of Diet in Renal Disease (MDRD), and prior aortic surgery. Anatomical characteristics included diameter and extent of the aneurysm, number of fenestrations in the device, and the diameter and length of the BeGraft stent grafts implanted.

### Preoperative Planning of Bridging Stents

All target arteries were analyzed on the preoperative computed tomography (CT) scan using a 3D workstation. A centerline was generated for each target artery and corrected manually when required. The curved planar reconstruction allowed analysis of the diameter of the target artery at the intended sealing zone, and the distance from its origin to the first division branch. The seal zone was defined as the length of stent in the target vessel with vessel wall apposition and was planned to be ≥10 mm. Using both parameters, the bridging stent’s diameter and length were determined preoperatively, with no oversizing of the stent diameter, and no coverage of divisional branches of the target vessel. All patients received single antiplatelet therapy ASA 75 mg (the standard dose in France) at the time of the procedure. During the procedure, to obtain a target ACT >250 s, 100 units/kg of heparin were administered intravenously.

### Bridging Stent Implantation

The bridging stents were positioned so that 3 to 4 mm of the stent graft protruded into the aortic lumen and were inflated with a balloon to 10 bar. The sheath was advanced over the balloon during balloon deflation to facilitate advancement of the flaring balloon. The protruding segments were flared with 9 mm diameter, 20 mm length balloon (Mustang, Boston Scientific, Marlborough, Massachusetts) for the renal artery stents (implanted through 6 mm diameter fenestrations), and a 10 mm diameter, 20 mm length balloon for the mesenteric stents (implanted through 8 mm fenestrations). The proximal marker of the flaring balloon was advanced to the level of the fenestration markers and the balloon was inflated to 10 bar. Positive forward pressure was applied on the shaft of the balloon to position it horizontal to obtain a 360° flair of the aortic portion of the bridging stent. Intraoperative data on procedure length, fluoroscopy time, dose area product (gray-centimeters squared [Gy·cm^2^]), volume of contrast agent injected, stent-related events, and additional procedures were collected.

### Follow-up

Computed tomography scans were performed during the first postoperative month, at 1 year, and yearly thereafter. Doppler ultrasound was performed before discharge and at 6 months, 1 year, and yearly thereafter to determine stent status (occlusion/stenosis/patency). No specific postoperative antiplatelet regime was specified for bridging stent patency. During follow-up, adverse events were collected according to reporting standards.^2^ Renal insufficiency was defined as a GFR estimated with MDRD ≤60 mL/min and renal impairment as a postoperative decrease in GFR of more than 20% compared with the preoperative GFR. Follow-up was defined as early during the first 30 postoperative days, and late thereafter. The study was approved by the research ethics committee and signed informed consent was obtained from all patients.

**Figure 1.**
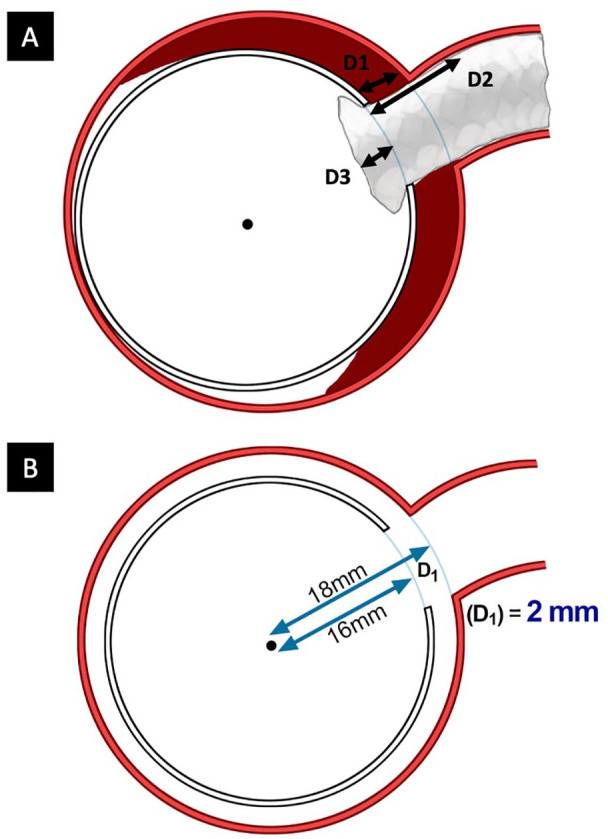
Distances measured for each fenestration/target vessel (A). Measurement method for D1 using vessel centerline (B). D1, fenestration to the origin of the target vessel; D2, fenestration to the seal zone in the target vessel; D3, fenestration to the end of bridging stent in aortic stent graft lumen.

On the postoperative CT and on the last available follow-up CT, the following distances were measured along the target vessel centerline for each fenestration/target vessel (Figure 1):

1. Fenestration to the origin of the target vessel (D1)
2. Fenestration to the seal zone in the target vessel (D2)
3. Fenestration to the end of bridging stent in aortic stent graft lumen (D3)

### Statistical Analysis

Continuous variables are expressed as median with interquartile ranges (Q1–Q3). Categorical variables are presented as percentages. Survival analyses were represented using the Kaplan-Meier method. Follow-up index was defined as the ratio between the investigated follow-up period and the theoretically possible follow-up period to the study end date.^3^ Statistical analyses were run using R statistical software.

## Results

### Preoperative Demographics

Thirty-nine consecutive patients (38 men) were enrolled and 101 BeGraft Peripheral Stent Grafts were implanted. The median age was 69.0 years (66.0–74.5 years) and the median aneurysm diameter was 60 mm (54.5–67.0 mm). Twenty-nine patients (75%) had a maximum diameter >55 mm (median diameter: 64 mm [59–70 mm]), and 10 patients had a maximum diameter >50 mm and <55 mm with rapid growth (median diameter: 52 mm [51–53 mm]). Aneurysms were juxtarenal and pararenal (19/39, 48.1%), type 4 TAAA (3/39, 7.7%), type 1, 2, and 3 TAAA (7/39, 17.8%), type 5 TAAA (4/39, 10.2%), and 15.4% (6/39) had a type I endoleak following a previous EVAR. Among the 39 patients, 3 were treated for TAAA post-dissection aneurysms. Patient characteristics are detailed in Table 1. Prior aortic surgery had been performed in 41% of patients (16/39). The procedure data have been previously described.^1^

**Table 1.** Patient Characteristics.

Medical history	Total^a^ n=39
Gender: male	38 (97.4)
Age (years)	69.0 (66.0–74.5)
Body mass index (kg/m²)	28.9 (26.1–32.0)
High blood pressure	35 (89.7)
Dyslipidemia	30 (76.9)
Diabetes mellitus	9 (23.1)
Chronic obstructive pulmonary disease	13 (33.3)
Prior myocardial infarction	16 (41.0)
Renal insufficiency	10 (25.6)
Dialysis	1 (2.5)
Previous smoker	24 (61.5)
Current smoker	11 (28.2)
Sleep apnea syndrome	8 (20.5)
American Society of Anesthesiologists score ≥3	34 (87.2)
Prior aortic surgery	16 (41.0)
Prior thoracic endograft	6 (15.4)
Prior abdominal endograft	6 (15.4)
Prior abdominal aortic surgery	4 (10.2)
Anatomical characteristics	
Type I endoleak after EVAR	6 (15.4)
Pararenal aneurysm	10 (25.6)
Juxtarenal aneurysm	9 (23.1)
Thoracoabdominal aneurysm	
Type 1	2 (5.1)
Type 2	3 (7.7)
Type 3	2 (5.1)
Type 4	3 (7.7)
Type 5	4 (10.2)
Preoperative diameter (mm)	60.0 (54.5–67.0)

Abbreviation: EVAR, endovascular aneurysm repair.

a Data are presented as median (Q1–Q3) or n (%).

### Bridging Stent Data

Among the 101 BeGraft stent grafts delivered, 55 were implanted in mesenteric arteries (22 in the coeliac trunk, 31 in the superior mesenteric artery [SMA], and 2 in the hepatic or splenic artery) and 46 into renal arteries (24 right and 22 left). The renal artery diameters were 5, 6, 7, and 8 mm in 9, 7, 26, and 4 patients, respectively. Mesenteric arteries were stented with 7 , 8 , 9 and 10 mm diameter BeGraft stent grafts. Short lengths (18 mm) were only required in 2 renal arteries. Long-length (37 or 38 mm) stents were required in 6 target vessels, including 2 renal arteries. All other target vessels were stented with 22-to-23 or 27-to-28 mm long BeGraft stent grafts. One patient was treated with a 5 to 18 mm BeGraft device as a suitable V12/iCast device was not available. Details are presented in Table 2. Two stents required additional angioplasty, 2 an additional covered stent, and 1 an additional nitinol stent. In one case, the intra-aortic portion of the bridging stent was shown to be kinked on completion cone beam CT. The kink was most likely caused during insertion of the delivery system of the bifurcated component after bridging stent had been deployed. The BeGraft was successfully catheterized antegrade and an additional covered stent (BeGraft) was deployed to treat the kink; we chose to reline the stent in case a fabric tear was also present.

**Table 2.** Stent Data.

Diameter (length)	CT	SMA	Other visceral arteries	Renal arteries
N	22	31	2	46
5–18	0	0	0	1
5–22	0	0	0	2
5–28	0	0	0	6
6–28	0	0	0	6
6–38	0	0	0	1
7–18	0	0	0	1
7–23	2	0	1	13
7–27	4	7	1	11
7–37	0	1	0	1
8–27	3	10	0	4
9–27	8	7	0	0
10–27	3	5	0	0
10–37	2	1	0	0

Abbreviations: CT, computed tomography; SMA, superior mesenteric artery.

### Early Outcome (≤30 Postoperative Days)

These data are included in Spear et al.^1^ To summarize, the median intensive care and hospital stays were 1 (1–4) and 10 (8–15) days, respectively. No early mortality occurred. BeGraft stent graft patency was 99% (100/101). There were 8 secondary procedures for access complications, bowel ischemia, hemorrhage from wire perforation, and embolic complications. The wire perforated ileal branches and caused acute hemorrhage, requiring emergent laparotomy to control the bleeding. The bowel ischemia occurred following an SMA dissection probably caused by a wire injury. The patient was treated with bowel resection and SMA stenting with a self-expanding nitinol stent and was alive at last follow-up (40 months). One secondary procedure (2.6%) was related to a BeGraft stent complication that required complementary stenting; the renal bridging stent was depicted thrombosed on immediate postoperative CT scan and the patient underwent a secondary procedure to try to restore patency of the renal bridging stent but this was unsuccessful. In the same patient, the contralateral renal bridging stent was also thrombosed at 2 month follow-up. Both renal artery stents were 5 mm in diameter. Two patients required postoperative temporary dialysis (2/39, 5.1%).

### Late Outcome (>30 Postoperative Days)

The median follow-up was 33 months (IQ25 17–IQ75 36). The follow-up index of the study was 0.85 (0.42–0.95). During follow-up, 11 patients died (28%), 6 are known to have died from non–aneurysm-related causes, and in 5 the cause of death was unknown. One patient was lost to follow-up after 13 months; no death on the National Death Registry was registered until the end of the study for this patient. All BeGraft stents were patent at last follow-up before death except in the patient previously described that had both renal stents thrombosed on the 2 month CT scan. Of the 11 deaths, 5 died in the first year of follow-up: 1 patient with renal artery thrombosis suffered from severe malnutrition and died at 3 months; 1 patient with cholesterol emboli syndrome died at 5 months from peritoneal infection; 3 patients died from stroke, hematological malignancy, and metastatic bowel cancer (not diagnosed preoperatively) at 2, 5, and 4 months postoperatively, respectively. Of the other 6 patients, death occurred at a median of 20 (minimum: 13; maximum: 25) months after the procedure. One of these patients died of neurosyphilis, the other causes of death are unknown. We estimated a mean±standard deviation (SD) event-free follow-up of 27±12.3 months and observed a mean±SD time to event of 13.2±14.9 months for primary patency, 7.33±11.0 months for secondary patency, and 9.8±13.6 months for target vessel instability (TVI).

Events that occurred during late follow-up are summarized in Table 3. Secondary procedures were performed in 7 patients (18%). Among these, 2 were performed on BeGraft stents, by endovascular means. One was to treat a left renal stent (7 × 23 mm) which was kinked and required an angioplasty at 31 months follow-up. The stent is still patent 8 months after the secondary procedure and 39 months after the index procedure. The second endovascular procedure was performed to treat a type Ic endoleak on a renal artery at 1 year follow-up. Additional stenting with a 7 × 27 mm BeGraft stent was performed 16 months after the initial procedure and is still patent at 35 months. Overall, the freedom from target vessel-related secondary procedure rate was 96% (97/101).

**Table 3.** Follow-up Data: Data are presented as median (Q1–Q3) or n (%).

Follow-up (months) Overall mortality (N=39)	33 (17–36) 11 (28)	
Secondary procedures (N=39)	Early (≤30 postoperative days)	Late (>30 postoperative days)
Overall	8 (20.5)	7 (18)
For stent complications	1 (2.6)	2 (5)
Endograft explant	0	1 (2.6)
Stent patency (N=101)	100 (99)	98 (97)

One patient underwent explantation at 17 months after the index procedure to treat an endoleak of unknown origin associated with symptomatic aneurysm growth. Once the aneurysm sac was opened, a type II endoleak was visualized. Only the bifurcated component was explanted, the fenestrated cuff was not removed. This patient had patent bridging stents after the explantation procedure at last follow-up.

**Figure 2.**
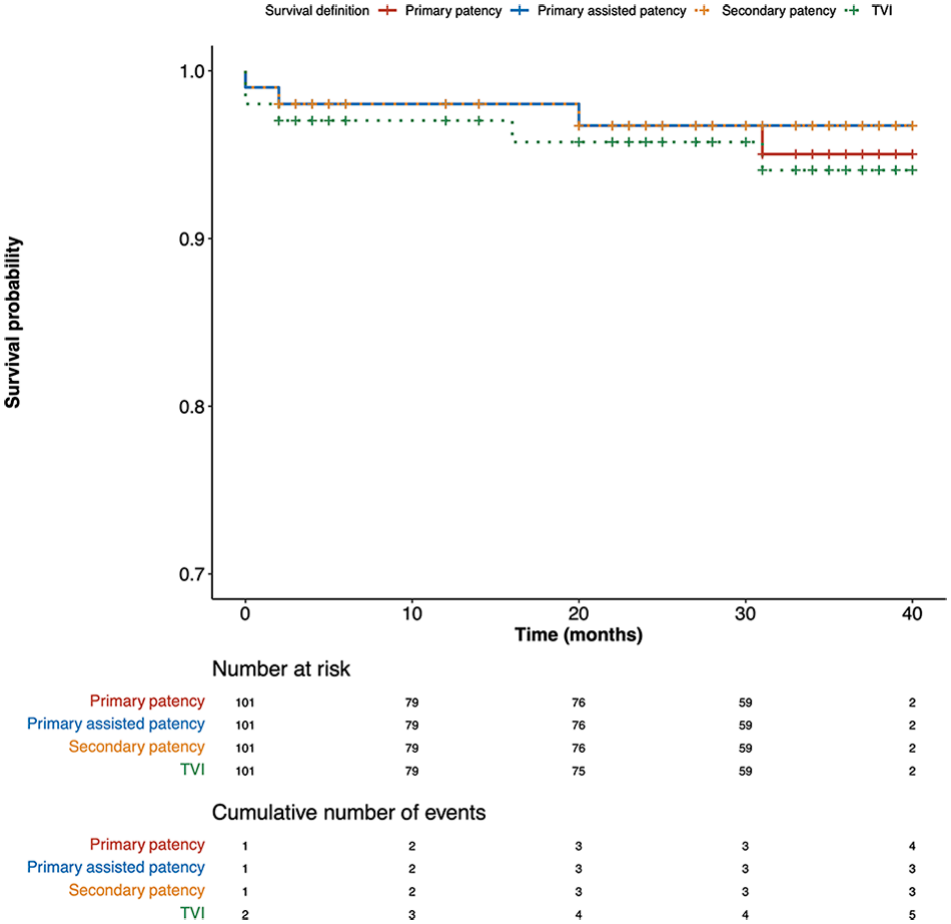
Analysis of primary patency, primary-assisted patency, secondary patency, and freedom from TVI. TVI, target vessel instability.

The overall patency rates were: primary patency: 96% (97/101); primary-assisted patency 97% (98/101); secondary patency 97% (98/101), and freedom from TVI 95% (96/101; Figure 2). Bridging stent thrombosis was diagnosed in 2 patients. The first patient has been described above. In the second patient, the left renal artery stent (7 × 27 mm) was found to be thrombosed at 20 months follow-up, with preserved renal function (50 mL/min/1.73 m²) on last follow-up. No other adverse events occurred in this patient.

The combined endpoint of “secondary procedure and thrombosis” was found in 5% (5/101) of the BeGraft stents implanted at a median follow-up of 33 months. All events occurred on stents implanted in renal arteries.

The measures performed to evaluate the fenestration/target vessel interface are shown in Table 4.

**Table 4.** Measurements Performed on Initial and Last Follow-up CT.

	Mean	Median	Minimum	Maximum
LRA				
LRA fenestration to origin of LRA	0.7/0.6	0/0	0/0	4.6/4.2
LRA fenestration to sealing zone in the LRA	3.0/2.8	2.4/2.2	0.8/1.3	11.2/7.5
Distance bridging stent extends into the graft from LRA fenestration	4.1/3.9	4.1/3.8	1.8/2.1	7.7/7.5
RRA				
RRA fenestration to origin of RRA	0.8/0.8	0/0	0/0	12/17.2
RRA fenestration to sealing zone in the RRA	3.1/3.2	2.4/2.4	0.6/1	13.3/17.2
Distance bridging stent extends into the graft from RRA fenestration	3.7/3.8	3.6/3.7	0.8/1.5	8.9/9.2
SMA				
SMA fenestration to origin of SMA	0.7/0.6	0/0	0/0	10.7/10.2
SMA fenestration to sealing zone in the SMA	3.3/3.9	2.5/2.4	0.7/0.8	14/31.2
Distance bridging stent extends into the graft from SMA fenestration	4.1/3.9	4.1/3.6	1.1/0.9	7.9/7.3
CT				
CT fenestration to origin of CT	0.5/0.3	0/0	0/0	11.7/7.5
CT fenestration to sealing zone in the CT	2.9/2.7	2.4/2.5	1.4/0.8	13.7/9.8
Distance bridging stent extends into the graft from CT fenestration	3.7/3.9	3.7/3.9	0.8/1.8	7.3/6.8

Abbreviations: CT, celiac trunk; LRA, left renal artery; RRA, right renal artery; SMA, superior mesenteric artery.

*Fenestration to origin of the target vessel (D1)*: The large majority of endografts were designed to have wall apposition between the fenestration and the origin of its target vessel (ie, median/mean distance from fenestration to origin of the target vessel = 0 mm) and this is reflected in the data presented. However, 1 patient had a gap between the RRA and the SMA fenestrations and the respective target vessel of >10 mm at implantation. This is the only patient that experienced an increase in this distance during follow-up; it was not associated with endoleak or aneurysm growth.

*Fenestration to seal zone in the target vessel (D2)*: In the majority of patients, the seal between the bridging stent and the target vessel started at 2.2 to 2.5 mm into the target vessel and extended for ≥10mm.

*Fenestration to end of bridging stent in aortic stent graft lumen (D3)*: The median length of bridging stent protruding into the aortic lumen ranged from 3.6 to 4.1 mm and was very constant when comparing all fenestrations and when comparing both time points.

## Discussion

The data from this study demonstrate favorable midterm outcomes of the BeGraft bridging stent used in fEVAR, with primary patency: 96% (97/101), primary-assisted patency 97% (98/101); secondary patency 97% (98/101), and freedom from TVI 95% (96/101). This is in keeping with the literature which demonstrates fEVAR for complex TAAAs has evolved significantly over the past years, with relatively high target vessel (TV) patency rates, which are currently reported as 92% to 99% in midterm to long-term follow-up.^4–7^ Re-interventions such as for type III endoleak, stenosis, and occlusion occur during follow-up in 8% to 26% of the patients and are a significant issue after fEVAR.^8,9^

The choice of bridging stent and the respective target vessel outcome for directional branches have been reported previously by multiple authors.^10–12^ Limited data exist on the outcomes of reinforced fenestrations stented with different bridging stents. Most publications report a composite outcome on a per vessel basis, which includes both directional branches and reinforced fenestrations; therefore, specific outcome comparisons are difficult, given the heterogeneity of the reported data; a thorough analysis would require randomized and prospective data collection with comparison of standardized outcomes.^10^ In general, renal artery-related adverse events are more common than mesenteric vessel-related adverse events,^11,13^ which was also found in this series. The kidneys undergo a considerable amount of displacement in response to respiratory motion, with a mean displacements of 20 mm^3^ reported.^14^ They also typically have a smaller starting diameter compared with the mesenteric vessels and a higher resistance to end-organ perfusion compared with the low resistance systems of the mesenteric and hepatic circulation. Small target vessels are difficult to manage in general (particularly <6 mm) due to the inherent difficulties in accurate planning and alignment of the aortic main-body stent graft. The small diameters of the bridging stents used are associated with low blood flows and therefore the risk of occlusion.^15^ Our preference is to use the BeGraft in lengths ≥22 mm and we have very limited experience of the use of the BeGraft Peripheral Stent Graft in 18 mm lengths. The 18 mm device is only used in short renal trunks and should be an exception because of the risk of inadequate seal.

The durability of fEVAR is largely dependent on the performance of bridging stent grafts, for which currently no dedicated stent is available on the market. The Gore Viabahn (VBX) balloon-expandable covered stent (W. L. Gore & Associates, Flagstaff, Arizona) was designed for use during branched repair in the Gore Excluder Thoracoabdominal Branched Endoprosthesis study. Although it is approved for use within the physician-sponsored investigational device exemption trial, VBX is not currently Food and Drug Administration approved for commercial use as a bridging covered stent for branched or fenestrated endovascular aortic repair.

The BeGraft Peripheral Stent Graft (Bentley InnoMed GmbH, Hechingen) is a balloon-expandable covered stent with a great amount of flexibility. It has been used “off-label” as a bridging stent graft in endovascular aortic aneurysm repair with favorable short-term outcomes.^1,6^ We chose to use the BeGraft device in our practice because of the profiles available (6 Fr for most stent diameters is a huge technical benefit) and because of the 8, 9, and 10 mm diameters with 27 mm lengths, which is well suited to the SMA and coeliac trunk. Bentley offers 2 stent systems, the BeGraft Peripheral and the BeGraft Peripheral PLUS, which has greater radial force compared with the former. We use the BeGraft Peripheral Stent Graft for fEVAR and the PLUS for branched devices, the rationale being, that in fEVAR the bridging stent does not cross the sac lumen, as it goes directly from the fenestration into the target vessel, whereas with branched devices, most of the bridging stent is within the sac lumen, with no external support and therefore requires a device with high circumferential radial force. A range of in vitro studies have been undertaken to study the biomechanical properties of current bridging stents.^16–18^ In these studies, the BeGraft showed no stent fracture or alteration in the expanded PTFE (ePTFE) coverage on flaring, and similar pull-out forces and resistance to dislocation of the device at 150% of the stent diameter to the Advanta (V12/iCast) device. These properties, when assessed in the BeGraft PLUS device, surpassed both the BeGraft and the Advanta devices.

The ideal bridging stent would likely have a low profile, good flaring capability, high radial force, flexibility, resistance to thrombosis, and intimal hyperplasia, with fabric that is resistant to tearing. The inclusion of a balloon for flaring on the delivery system of the device would improve efficiency during the procedure and reduce the risk of complications caused by device exchange. Care must also be exercised when introducing delivery systems, for example for the bifurcated component, after the bridging stent had been deployed; potential adjuncts are use of short tips for the delivery systems or stenting using alternate access after the bifurcated device and iliac legs have been deployed.

Imaging plays a crucial role in the planning, intraoperative guidance, and postoperative surveillance of fenestrated endovascular aortic repair.^2^ Careful preoperative planning has an important influence on branch-related outcomes. In our practice, the target vessel centerline is calculated and this information, together with the position of the first branch and the global 3-dimensional structure of the branch, is used to determine the ideal bridging stent length, position, and seal zone. This thorough analysis is intended to reduce the incidence of end-organ ischemia and injury to the target vessel. As shown in this series, selecting the appropriate bridging stent is associated with very consistent and secure positioning of the stents through the fenestrations during follow-up (Table 4). We use non-contrast-enhanced cone beam CT intraoperatively to provide 3-dimensional images of the stent conformation in the endovascular repair prior to completion of the procedure. This allows problems with the repair such as stent malignment or disconnection to be seen early and treated under the same anesthetic as the primary procedure. The good secondary durability seen in this series is a reflection of a strict and comprehensive imaging surveillance protocol used, which allows early detection of problems and timely re-intervention, preventing potential major adverse events. We believe that appropriate planning of the custom-made endograft also plays a key role. As reported in Table 4, most fenestrations abutted the origin of their respected TV, which provided a stable platform and thus less constraint on the bridging stent over time. Further evaluation of the target vessels in terms of preoperative measurement of vessel angulation, stenosis, and calcification is of interest, particularly in terms of the influence these have on the performance and durability of the bridging stents, and are limitations of the current study and the aim of future work.

## Conclusion

The data from this study demonstrate the early favorable outcomes of the BeGraft bridging stent used in fEVAR are confirmed during midterm follow-up, with low rates of endoleak and stent occlusion. Strict and comprehensive imaging surveillance allows early detection of problems and timely re-intervention, preventing potentially major adverse events.
